# Effects of percutaneous endovascular angioplasty for severe stenosis or occlusion of subclavian artery

**DOI:** 10.1038/s41598-024-65302-y

**Published:** 2024-06-21

**Authors:** Tong-Yuan Zhao, Gang-Qin Xu, Jiang-Yu Xue, Dong-Yang Cai, Bo-Wen Yang, Yue-Yang Ba, Chen-Yi Feng, Tian-Xiao Li, Bu-Lang Gao, Zhong-Can Chen

**Affiliations:** Stroke Center, Cerebrovascular Disease Hospital, Henan Provincial People’s Hospital, 7 Weiwu Road, Zhengzhou, 450003 Henan Province China

**Keywords:** Severe subclavian artery stenosis ≥ 70%, Occlusion, Percutaneous endovascular angioplasty, Stent deployment, Balloon expansion, Diseases, Health care, Medical research, Neurology

## Abstract

To investigate the effect and safety of percutaneous endovascular angioplasty (PEA) with optional stenting for the treatment of severe stenosis or occlusion of subclavian artery, patients with severe stenosis ≥ 70% or occlusion of subclavian artery treated with PEA were retrospectively enrolled. The clinical data were analyzed. A total of 222 patients were retrospectively enrolled, including 151 males (68.0%) and 71 females (32.0%) aged 48–86 (mean 63.9 ± 9.0) years. Forty-seven (21.2%) patients had comorbidities. Subclavian artery stenosis ≥ 70% was present in 201 (90.5%) patients and complete subclavian occlusion in 21 (9.5%) cases. Angioplasty was successfully performed in all (100%) patients. Balloon-expandable stents were used in 190 (85.6%) cases, and self-expandable stents in 20 (9.0%) cases. Only 12 (5.4%) cases were treated with balloon dilation only. Among 210 patients treated with stent angioplasty, 71 (33.8% or 71/210) cases underwent balloon pre-dilation, 139 (66.2% or 139/210) had direct deployment of balloon-expandable stents, and 2 (1.0% or 2/210) experienced balloon post-dilation. Distal embolization protection devices were used in 5 (2.3% or 5/222) cases. Periprocedural complications occurred in 3 (1.4%) patients, including aortic dissection in 2 (0.9%) cases and right middle cerebral artery embolism in 1 (0.5%). No hemorrhage occurred. Among 182 (82.0%) patients with 6-month follow-up, restenosis > 70% occurred in 1 (0.5%) patient, and among 68 (30.6%) patients with 12-month follow-up, restenosis > 70% took place in 11 (16.2%) patients. Percutaneous endovascular angioplasty can be safely and efficiently performed for the treatment of severe stenosis ≥ 70% or occlusion of subclavian artery.

## Introduction

The incidence of subclavian artery stenosis may vary significantly in different groups of patients, with 0.8–2% in the common population but up to 8.5% in patients with coronary artery disease^1–4^. The most common reason for subclavian artery stenosis is atherosclerosis, with the commonest location of stenosis proximal to the origin of the vertebral artery. Because of the subclavian artery stenosis at this location^4–7^, collateral circulation from the contralateral vertebral artery or the Willis circle is frequently insufficient and causes relevant ischemic symptoms of the brain, back and upper limbs. For revascularization of the stenosis, endovascular and surgical therapeutic approaches are both available, with low risks of severe complications^8^. As percutaneous transluminal angioplasty has been applied increasingly clinically, it has become the first choice of strategy for management of subclavian artery stenosis because of the low complication rates and durable effects of treatment^1,4,8–14^. Currently, not many studies with a large sample of patients treated with endovascular angioplasty of subclavian artery stenosis have been reported, and issues related to the endovascular angioplasty including operation skills, use of stents, and selection of appropriate patients have been less clearly investigated, which may thus serve as a greater challenge for future application of the endovascular angioplasty in this field. This study was consequenlty performed to further explore how to better apply percutaneous endovascular angioplasty for subclavian artery stenosis so as to improve the treatment effectiveness, reduce postoperative complications, and ultimately benefit patients.

## Materials and methods

### Subjects

This retrospective single-center study was approved by the ethics committee of Henan Provincial People’s Hospital, and informed consent was waived by the same ethics committee because of the retrospective study design. All methods were conducted in accordance to relevant guidelines and regulations. Between January 2014 and January 2021, patients with subclavian artery stenosis or occlusion treated with percutaneous endovascular angioplasty and optional stenting were retrospectively enrolled. The inclusion criteria were patients with severe symptomatic subclavian artery stenosis ≥ 70% or occlusion confirmed by imaging examination, vertebrobasilar artery steal symptoms (such as dizziness, diplopia, syncope, etc.), chronic upper limb ischemia (such as weak arm force, rest pain, numbness or ulcer), and involvement of the internal mammary artery for blood supply to the heart leading to angina. Patients with asymptomatic severe subclavian artery stenosis ≥ 70% were also enrolled if any one of the following conditions was present: the affected internal mammary artery being selected for coronary artery bypass grafting, myocardial ischemia caused by proximal subclavian artery stenosis after coronary artery bypass grafting using the affected internal mammary artery, use of an artificial arteriovenous fistula on the affected side for dialysis treatment in patients with hemodialysis, and bilateral subclavian artery stenosis resulting in inaccurate measurement of the actual central blood pressure through upper limb artery. Patients without the above indications were excluded for endovascular angioplasty for subclavian artery stenosis. The indications for revascularization of the subclavian artery were determined according to individual patient factors, including symptoms, accompanying medical conditions, surgical risks, available vascular pathways, life expectancy, and patient preferences.

### Endovascular procedure

The endovascular procedure was performed under local or general anesthesia. Three days prior to the procedure, dual antiplatelet therapy with aspirin (100 mg/day) and clopidogrel (75 mg/day) was administered in all patients. During the whole procedure, systemic heparin was administered to maintain an activated coagulation time of 250–300 s. After successful puncture of the right femoral artery using the Seldinger puncture technique, an 8F arterial sheath (Cordis, Johnson&Johnson, Bridgewater, NJ, USA) was inserted before a guide wire was used to introduce an 8F guiding catheter (Cordis, Johnson&Johnson, Bridgewater, NJ, USA) to the ostium of the subclavian artery for angiography. The location, length, and degree of the subclavian artery stenosis were recorded and evaluated. A micro-guide wire (Cordis, Johnson&Johnson, Bridgewater, NJ, USA) was navigated across the stenosis or occlusion site before an appropriate balloon catheter (Cordis, Johnson&Johnson, Bridgewater, NJ, USA) was sent to the stenosis or occlusion site for dilation. A proper balloon-expandable (SD, Boston Scientific, Boston, MA, USA) or self-expandable stent (Cordis, Johnson&Johnson, Bridgewater, NJ, USA) was selected for deployment at the lesion site. If the stent was not completely expanded or with a residual stenosis ≥ 30% after deployment, post-dilation was performed with a balloon to dilate the stent. After balloon expansion or stent deployment, angiography was performed to assess the patency and residual stenosis of the subclavian artery. A stent or a balloon was selected according to the equation: the stent or balloon diameter = (proximal normal arterial diameter + distal normal arterial diameter to the stenosis)/2.

After the procedure, dual antiplatelet therapy, including aspirin 100 mg/day and clopidogrel 75 mg/day, was continued for a duration of 3–6 months, followed by lifelong administration of a single antiplatelet agent (usually aspirin 100 mg/day) with the same dose. Before patient discharge, brachiocephalic vascular Doppler ultrasound examination was conducted in all patients to check the patency of the subclavian artery. Follow-up was conducted in all patients 6 and 12 months after the surgery at the outpatient department. During each visit, clinical examinations and Doppler ultrasound examinations were conducted. If there was suspected subclavian artery restenosis or occlusion, computed tomography angiography (CTA) was performed. In cases of subclavian artery restenosis exceeding 70% combined with vertebrobasilar artery dysfunction and steal syndrome and/or chronic upper limb ischemia, further intervention was necessary.

### Outcome evaluation

Short-term outcomes were evaluated according to the technical successful rate of angioplasty, periprocedrual complications, and 30-day mortality rates. Technical success of angioplasty was defined as patency of the subclavian artery without significant residual stenosis (less than 30% of the arterial inner diameter) after angioplasty, determined by intra- and postoperative angiography and Doppler ultrasound before discharge of the patient. Postoperative complications included any intraoperative accidents that required postoperative medical or interventional management or extended hospital stay. The 30-day mortality included all-cause deaths within 30 days after the angioplasty intervention.

Long-term outcomes included patency of the subclavian artery, occurrence of cardiac and neurological events, and all-cause mortality during follow-up. Restenosis was defined based on narrowing of the arterial lumen as recurrence of arterial stenosis ≥ 70%. Any events involving the heart, such as ventricular fibrillation, complete heart block, myocardial infarction, cardiac arrest, or cardiac death, were recorded as the cardiac event. Neurological events included any events involving the brain, such as transient ischemic attack, ischemic stroke, intracranial hemorrhage, coma, or brain death. Any cardiac and neurological events, as well as all-cause deaths, were recorded on medical records upon admission or through a death certificate in the event of death.

### Data collection

The types of aortic arch were divided into three types: type I with all supra-aortic vessels originating at the same level in a straight line, type II with the innominate and left common carotid arteries originating below the left subclavian artery, and type III with all supra-aortic arteries originating below the straight line with an acute angle formed between the artery origin and aortic arch^15,16^. The following data were collected: patient’s age, sex, comorbidities, clinical symptoms, imaging presentations, dual antiplatelet therapy, type of aortic arch, access cite, balloon expansion, stent deployment, technical success, periprocedural complications, surgical time, X-radiation time, follow-up time, recurrence of arterial stenosis and symptoms, short- and long-term outcomes.

### Statistical analysis

The SPSS software (Version 22.0, IBM, Chicago, IL, USA) was used for the statistical analysis of this study. Continuous measurement data were presented in means and standard deviations if in the normal distribution and tested with the t test or in medians and interquartile ranges if not in the normal distribution and tested with the Mann Whitney U test. Categorical data were presented in frequencies and percentages and tested with the Chi square test. The significant *P* was set at < 0.05.

## Results

A total of 222 patients with severe subclavian artery stenosis ≥ 70% or occlusion were enrolled (Table 1), including 151 males (68.0%) and 71 females (32.0%), with a male to female ratio of approximately 2:1 and an age range 48–86 (mean 63.9 ± 9.0) years. Subclavian artery stenosis ≥ 70% (70–99%, mean 92%) was present in 201 (90.5%) patients and complete subclavian occlusion in 21 (9.5%) cases. Subclavian artery stenosis was 70–80% in 55 (24.8%) patients, 80–90% in 106 (47.7%), and over 90% in 40 (18.0%).

**Table 1:** The baseline data of patients.

Variables	Data
Sex (n, %)	
Male	151 (68.0%)
Female	71 (32.0%)
Age (y)	48–86 (63.9 ± 9.0)
Subclavian artery stenosis (n, %)	
70–80%	55 (24.8%)
80–90%	106 (47.7%)
> 90%	40 (18.0%)
Subclavian artery occlusion (n, %)	
Complete occlusion	21 (9.5%)
Clinical conditions (n, %)	
Hypertension	151 (68.0%)
Diabetes mellitus	52 (23.4%)
Hyperlipidemia	128 (57.7%)
Smoking	44 (19.8%)
Intracranial aneurysms	3 (1.4%)
Vertebral artery stenosis	21 (9.5%)
Carotid artery stenosis	21 (9.5%)
Innominate artery stenosis	1 (9.5%)
Middle cerebral artery stenosis	1 (9.5%)
Tumor	1 (9.5%)
Type of aortic arch	
Type I	45 (20.3%)
Type II	122 (55.0%)
Type III	55 (24.7)

Except for 1 (0.5%) case of arteritis and 1 (0.5%) case of tumor compression, all patients had significant high risk factors of atherosclerosis, including hypertension in 151 (68.0%) cases, diabetes mellitus in 52 (23.4%), hyperlipidemia in 128 (57.7%), and smoking in 44 (19.8%). All patients experienced symptoms related to subclavian stenosis or occlusion, with dizziness being the most common in 78 (35.1%) cases, episodic syncope in 15 (6.8%), episodic diplopia in 5 (2.2%), upper limb ischemic symptoms of pain, numbness, and weakness in 48 (21.6%), cold skin in 14 (6.3%), and pulse disappearance in 55 (24.8%). Forty-seven (21.2%) patients had comorbidities of vascular diseases in the head and neck: 3 (1.4%) patients with intracranial aneurysms, 21 (9.5%) with vertebral artery stenoses, 21 (9.5%) with carotid artery stenoses, 1 (0.4%) with an innominate artery stenosis, and 1 (0.4%) with a middle cerebral artery stenosis.

**Figure 1:**
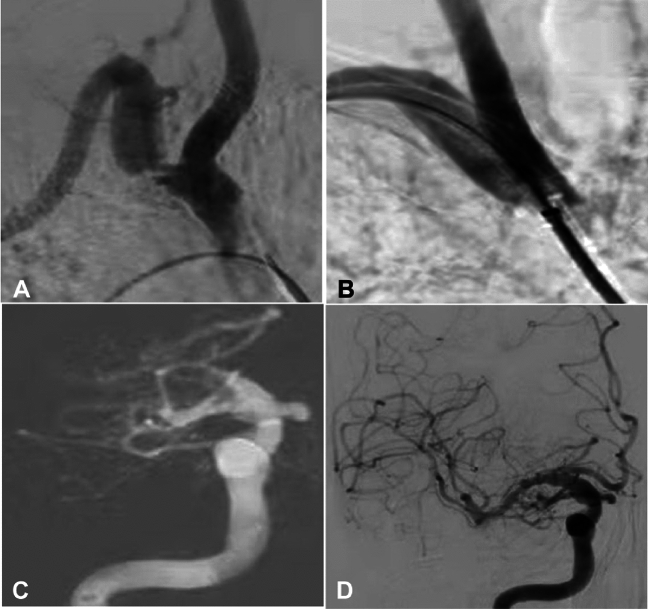
Percutaneous endovascular angioplasty was performed in a male patient aged in their 80 s with subclavian artery steal syndrome and paroxysmal syncope. (**A**) The right subclavian artery stenosis was approximately 95% on agngioraphy. (**B**) After deployment of a balloon-expandable stent at the stenotic segment, the stenosis disappeared. (**C**) Embolism occurred in the right middle cerebral artery causing disappearance of the right middle cerebral artery. (**D**) After immediate endovascular mechanical thrombectomy, patency of the right middle cerebral artery was restored, resulting in the thrombolysis in cerebral infarction score of 2b.

**Figure 2:**
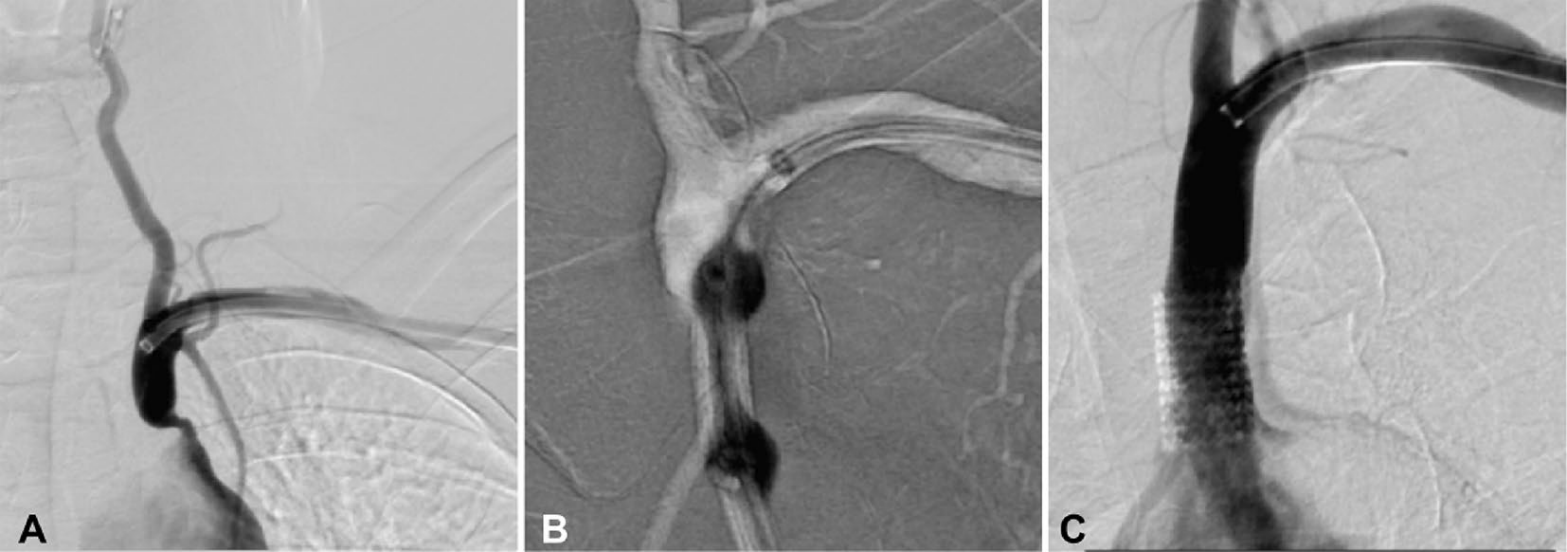
Left subclavian artery stenosis treated with a balloon-expandable stent in a female patient in their 60 s with intermittent dizziness for more than half a year, weak left pulse, and a 30 mmHg difference in blood pressure between bilateral brachial arteries. (**A**) Subclavian angiography through the left radial artery access demonstrated a severe stenosis of approximately 90% of the left subclavian artery. (**B**) A 8 mm × 17 mm balloon-expandable stent was precisely positioned at the stenotic location and deployed to repair the stenosis. (**C**) After stent angioplasty, angiography revealed patent stent with a residual stenosis < 30%.

**Figure 3:**
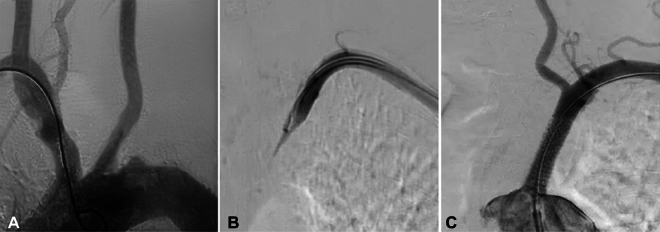
Left subclavian artery stenosis treated with a self-expandable stent in a male patient in their 60 s with dizziness for over 1 year, no pulse and resting pain in the left upper limb for over 6 months. (**A**) Aortic arch angiography through the left radial artery revealed occlusion of the left subclavian artery, with a blunt proximal end of the occluded segment. (**B**) The occlusion was explored with a guide wire, and angiography at the ostium of the left subclavian artery showed a cavity at the distal end of occlusion. (**C**) The occlusion was expanded with a balloon before a 7 mm × 17 mm self-expandable stent was positioned at the stenosis and deployed. Post-deployment showed patent stent with a residual stenosis < 30%.

Angioplasty was successfully performed in all 222 (100%) patients, resulting in a residual stenosis < 30% (Table 2 and Figs. 1, 2 and 3), with local anesthesia in 202 (91.0%) and general anesthesia in 20 (9.0%) patients. General anesthesia was performed because of concurrent endovascular treatment of other diseases or being unable to cooperate or tolerate the local anesthesia treatment. Femoral access was obtained in 207 (93.2%) patients while access through both the femoral and radial arteries in 15 (6.8%) patients. Angioplasty was performed on the right side in 66 (29.7%) cases, left in 154 (69.4%), and both sides in 2 (0.9%), with a left to right ratio of approximately 3:1 for the angioplasty. Balloon-expandable stents were used in 190 (85.6%) cases with stenting on the right side in 59 (31.1% or 59/190) cases, with a stent diameter of 5–10 mm, and self-expandable stents in 20 (9.0%) cases with stenting on the right side in five cases (25% or 5/20) and an average stent diameter of 7.9 mm. Only 12 (5.4%) cases were treated with balloon dilation only. Among 210 patients treated with stent angioplasty, 71 (33.8% or 71/210) cases underwent balloon pre-dilation, 139 (66.2% or 139/210) underwent direct deployment of balloon-expandable stents, and 2 (1.0% or 2/210) experienced balloon post-dilation. Distal embolization protection devices were used in 5 (2.3% or 5/222) cases. No significant (*P* = 0.34) difference was found in the access site or type of stents between the right and left side of treatment.

**Table 2:** Surgical data.

Variables	Data
Anesthesia (n, %)	
Local	202 (91.0%)
General	20 (9.0%)
Access (n, %)	
Femoral artery	207 (93.2%)
Radial artery	15 (6.8%)
Disease (n, %)	
Subclavian stenosis	201 (90.5%)
Subclavian occlusion	21 (9.5%)
Residual stenosis (n, %)	
0–25% (16%)	222 (100%)
Side of angioplasty (n, %)	
Right	66 (29.7%)
Left	154 (69.4%)
Both sides	2 (0.9%)
Technical success rate	100%
Angioplasty mode (n, %)	
Balloon-expandable stents	190 (85.6%)
Self-expandable stents	20 (9.0%)
Balloon expansion alone	12 (5.4%)
Treatment mode in stent angioplasty (n, %)	
Balloon pre-dilation	71 (33.8%)
Direct balloon-expandable stent angioplasty	139 (66.2%)
Balloon post-dilation	2 (1.0%)
Distal embolization protection devices (n, %)	5 (2.3%)

Type I aortic arch was present in 45 (20.3%) patients, type II in 122 (55.0%), and type III in 55 (24.75) (Table 1). The fluoroscopy time and amount of contrast medium used were 13.4 ± 3.2 min and 150 ml, respectively, for both types I and II aortic arch, significantly smaller than those for type III aortic arch (18.3 ± 4.7 min and 220 ml, respectively). After angioplasty, the residual stenosis was 0–25% (16%), which was significantly (*P* < 0.001) smaller than the stenosis degree before treatment. Periprocedural complications occurred in 3 (1.4% or 3/222) patients, including aortic dissection in 2 (0.9% or 2/222) cases and right middle cerebral artery embolism in 1 (0.5% or 1/222) (Table 3 and Fig. 1). No significant (*P* = 0.53) difference was found in the periprocedural complications between the right and left sides. No hemorrhage occurred. Two patients experienced aortic dissection, with chest pain presentation lasting for 1–3 days after angioplasty, and digital subtraction angiography 1 week later revealed healing in 1 (0.5% or 1/222) patient and no apparent changes in the aortic dissection in the other patient.

**Table 3:** Periprocedural complications and follow-up.

Variables	Data
Complications (n, %)	
Aortic dissection	2 (0.9%)
Right middle cerebral artery embolism	1 (0.5%)
Hemorrhage	0
Follow-up (n, %)	
Follow-up duration	6–19 (median 13)
Number of patients	205 (92.3%)
Restenosis > 70%	12 (5.9%)
Balloon angioplasty alone	2 (16.7%)
Balloon-expandable stents	7 (58.3%)
Self-expandable stents	3 (25%)
Symptoms	0
Mortality	0

Follow-up was conducted in 205 (92.3%) patients 6–19 (median 13) months after endovascular treatment. Restenosis over 70% occurred in 12 (5.9% or 12/205) patients including one patient who had arteritis-related stenosis treated with a Precise 8 mm × 30 mm stent (Cordis, Johnson&Johnson, Bridgewater, NJ, USA). Among 12 patients with restenosis > 70%, two (16.7% or 2/12) patients experienced balloon angioplasty alone, seven (58.3%) underwent stent-angioplasty with balloon-expandable stents, and three (25% or 3/12) were treated with self-expandable stents (Tables 3 and 4). No significant (*P* > 0.05) difference was detected in the restenosis rates between any two modalities of treatment (Table 4). All symptoms were significantly relieved, and no symptoms (transient attack or stroke) or mortality occurred at follow-up (Table 3).

**Table 4:** Restenosis at follow-up in patients treated with different modalities.

Variables	Balloon angioplasty alone	Ballon-expandable stents	Self-expandable stents	Total
Restenosis > 70%	2	7	3	12
No restenosis	10	183	17	210
Total	12	190	20	222

No significant (*P* > 0.05) difference existed between any two modalities.

## Discussion

In this study investigating the safety and effect of percutaneous endovascular angioplasty for the treatment of severe subclavian artery stenosis ≥ 70% or subclavian artery occlusion, it was found that percutaneous endovascular angioplasty was safe and efficient for the treatment of subclavian artery severe stenosis ≥ 70% or occlusion.

The current therapeutic approaches for chronic obstructive lesions of the subclavian artery mainly consist of extra-anatomic carotid-subclavian bypass, percutaneous endovascular angioplasty and stent deployment^1,4,5,7–14,17^. Conservative medication may play a role in patients with subclavian artery stenosis, but endovascular angioplasty had a 60% risk of reduction for hemodynamic subclavian stenosis compared with conservative medication (adjusted hazard ratio 0.4, 95% confidence interval 0.2–0.6, *P* < 0.0001) even though a significant (*P* = 0.3) symptomatic restenosis was detected at 42-month follow-up between patients treated with endovascular angioplasty and medication alone^18^. Thus, endovascular angioplasty may be reserved for severe symptomatic subclavian artery stenosis ≥ 70% or subclavian artery occlusion with critical ischemia, vertebrobasilar insufficiency, or peripheral emboli^18^.

There have also been studies comparing open surgery with endovascular treatment, resulting in a conclusion that open surgical repair may be the correct strategy for patients with a low risk of complications, unsuccessful endovascular revascularization, or a treatment plan using both open surgery and endovascular angioplasty^19–21^. Further, similar early outcomes have been demonstrated between open surgery and endovascular revascularization for subclavian artery atherosclerotic diseases in a systematic review and meta-analysis^22^. In this study with 297 patients treated endovascularly and 463 patients treated with open surgery^22^, no significant difference (*P* > 0.05) was revealed concerning the majority of early major outcomes (technical success, 30-day mortality, cardiac events, and central nervous system events), even though more patients undergoing open repair had an occlusion. Nonetheless, open surgery seemed to prevail over endovascular treatment regarding long-term primary patency despite no significant difference in the long-term survival and freedom from recurrent symptoms between the two groups of patients. Compared with endovascular angioplasty, open surgery may have a better patency as revealed by another study^23^, however, endovascular revascularization has been treated as the first-line treatment approach for subclavian artery stenosis or occlusion as significantly more experience has been gained. The advantages and disadvantages of stent deployment and balloon expansion alone have been analyzed^14^, and stent angioplasty was shown to be significantly better than balloon expansion alone in maintaining the 1-year patency (*P* = 0.004; 95% confidence interval, odds ratio 2.37 [1.32–4.26]) in treating subclavian artery stenosis without significant differences in the complication rates between the two procedures. This study is in favor of stent placement after angioplasty for successful recanalization of stenosed subclavian arteries and maintaining long-term patency without significant increase in the risk for major complications^14^.

In a study with 232 consecutive patients with subclavian artery stenosis or occlusion treated with percutaneous endovascular angioplasty^4^, severe complications took place in 9 (3.9%) patients, including 6 embolic complications (2.6%), 1 hemorrhagic (0.4%), and 2 dissections of the distal subclavian artery requiring immediate retrograde procedure (0.9%). Thirty days after the endovascular treatment, 85.4–98.7% patients were free from the symptoms caused by subclavian artery stenosis. In another study investigating endovascular therapy for subclavian artery occlusive diseases or stenosis involving the vertebral artery origin in 196 patients^24^, periprocedural complications occurred in 16 (8.2%) patients including death in one (0.5%) patient caused by endovascular procedure-related sepsis, stroke in one (0.5%) patient with the National Institutes of Health Stroke Scale of 3, transient ischemic attack in 2 (1.0%), plaque shift in 5 (2.6%) during the procedure, and vascular access site complications in 7 (4%). In one study enrolling 26 patients with subclavian artery stenosis or occlusion treated with endovascular angioplasty^8^, periprocedural complications included minor stroke in one patient, angioplasty-related arterial dissections in 3, and groin hematoma in 2, resulting in a complication rate of 23.1% (6/26). Another study involving 71 patients with subclavian artery stenosis treated with endovascular stenting reported a 5.6% complication rate (in four patients) with three complications of puncture side hematomas and one embolic stroke which resulted in procedure termination and technical failure^25^. In our study with 222 patients of subclavian artery stenosis ≥ 70% or occlusion, the success rate was 100%, and 3 (1.4%) patients experienced periprocedural complications with one (0.4%) severe complication of the right middle cerebral artery embolism.

Our study seemed to have a higher success rate and a lower complication rate, which may be related to the proficient skills in angioplasty, proper selection of treatment plan and endovascular devices. Good proficiency in angioplasty plays a crucial role in improving the success rate of endovascular revascularization. In our study, we had tried to simplify and standardize the operating procedures as much as possible and select an appropriate balloon-expandable stent to pass through the stenosis according to the arterial stenosis on angiography. After a guide wire and a stent were sent in place, balloon dilation was directly performed to deploy the stent. Studies have confirmed that fewer and simpler surgical steps will lead to a lower probability of complications under the same conditions^26–28^. The processes of puncture, balloon positioning, and stent release require sufficient skills and experience. In experienced hands, the proficiency level of skills is high, and the success rate of surgery can reach 100%. In one study with 49 patients of subclavian artery stenosis or occlusion treated with endovascular angioplasty^21^, all 21 patients with subclavian artery occlusion was successfully recanalized probably because of short occlusion time, a short occlusive segment, and existence of potential cavities in the occlusion segment. Nonetheless, the most important issue is still the surgical skills and proficiency level as stated below. A suitable access approach should be selected because the subclavian stent delivery system is relatively big and necessitates use of a larger access route like the femoral artery approach rather than the thinner radial artery. However, in some cases, the radial artery approach is preferred, such as in case of type III aortic arch accompanied by right subclavian artery stenosis or subclavian artery occlusion combined with the proximal end of occlusion being blunt and the distal end being rat-tailed. If necessary, multiple guide wires are used to stabilize the guiding catheter system by navigating a hard 0.014 “or 0.018” guide wire into the brachial or vertebral artery to stabilize the guiding catheter system without simultaneously affecting the delivery effect of the stent system. If the stenotic lesion involves the ostium of the vertebral artery, a guide wire should be preset in advance to avoid compression of subclavian plaques and subsequent vertebral artery occlusion. If feasible, the guiding catheter is passed through the stenotic segment before retracting it to deploy the stent. In this case, the stent position is adjusted and fixed inside the catheter before retracting the catheter system, which will result in less damage to the arterial wall and the atherosclerotic plaque. The coaxial technique with multiple catheters placed coaxially can not only increase the system support but also improve the ability to pass through the stenosis. A guiding wire and a guiding catheter can be placed in both the femoral artery and radial artery so as to guide the direction of the system and provide more valuable imaging results, which is important for revascularization of complete occlusion.

The selection and design of endovascular devices are crucial for the success of endovascular angioplasty. If a 6F 90 cm-long sheath is available, it should be chosen for the endovascular operation. A long sheath has a conical tip which causes less damage to the atherosclerotic plaque when passing through the stenotic segments, making it particularly suitable for revascularizing the left subclavian artery stenosis and deploying the stent using the system “retracting mode”. In recent years, new materials such as flexible intermediate catheters and long sheaths have made many previously extremely difficult procedures for Type III aortic arch much simpler, especially in combination of flexible catheters and VTK catheters, which has greatly reduced the difficulty of endovascular recanalization. Compared with balloon-expandable stents, self-expanding stents have better flexibility, better trackability in passing through difficult stenosis, and more stent models to choose from, making them a good choice. However, they also have the disadvantage of poor support. Therefore, proper balance of the appropriate stent type, patient age, medical history, and atherosclerotic risk factors can achieve better treatment outcomes. In addition, special treatment is recommended for cases with the stenosis of the right subclavian artery involving the brachiocephalic trunk. As shown in the case in the text (Fig. 1), plaque detachment and embolus formation led to ipsilateral middle cerebral artery embolism which is a serious complication. Therefore, placing a protective umbrella inside the right internal carotid artery is a good choice to protect the patient from suffering from severe cerebral complications.

Our study had some limitations, including the retrospective and single-center study design, a small sample of patients, Chinese patients enrolled only, no randomization or control, which may all affect the outcomes of the study. Future randomized, prospective, controlled, multicenter studies will have to be conducted to involve multiple races and ethnicities for better outcomes.

In summary, percutaneous endovascular angioplasty is safe and efficient for the treatment of subclavian artery severe stenosis ≥ 70% or occlusion even though further studies are necessary for the selection of optimal stents, technical skills, and proper indications so as to improve the treatment effect and prognosis and decrease the risk for periprocedural complications.

## Data Availability

The datasets used and/or analysed during the current study are available from the corresponding author on reasonable request.

## References

[1] Iared W, Mourao JE, Puchnick A, Soma F, Shigueoka DC (2022). Angioplasty versus stenting for subclavian artery stenosis. Cochrane Database Syst. Rev..

[2] Labropoulos N, Nandivada P, Bekelis K (2010). Prevalence and impact of the subclavian steal syndrome. Ann. Surg..

[3] Shadman R, Criqui MH, Bundens WP, Fronek A, Denenberg JO, Gamst AC (2004). Subclavian artery stenosis: Prevalence, risk factors, and association with cardiovascular diseases. J. Am. Coll. Cardiol..

[4] Wrotniak L, Kablak-Ziembicka A, Roslawiecka A, Musialek P, Bogacki P, Trystula M (2016). Resolution of ischemic symptoms after percutaneous angioplasty for a symptomatic subclavian artery stenosis. J. Vasc. Surg..

[5] Yamaguchi K, Funatsu T, Moteki Y, Nonaka T, Niwa A, Imanaka K (2023). Subclavian artery-carotid artery bypass for subclavian artery or common carotid artery severe stenosis or occlusion. Neurol. Med. Chir. (Tokyo).

[6] Shankar Kikkeri, N. & Nagalli, S. *Subclavian Steal Syndrome*. Treasure Island (FL) with ineligible companies. Disclosure: Shivaraj Nagalli declares no relevant financial relationships with ineligible companies (Statpearls, 2023).

[7] Wenkel M, Halloum N, Izzat MB, Ali-Hasan-Al-Saegh S, Duerr GD, Kriege M (2023). Long-term outcome of carotid-subclavian bypass in the management of coronary-subclavian steal syndrome. Vasc. Endovasc. Surg..

[8] Jahic E, Avdagic H, Iveljic I, Krdzalic A (2019). Percutaneous transluminal angioplasty of subclavian artery lesions. Med. Arch..

[9] Yamamoto N, Yamamoto Y, Yamaguchi I, Sogabe S, Miyamoto T, Shimada K (2022). Percutaneous transluminal angioplasty and stenting using an aspiration catheter. J. Neuroendovasc. Ther..

[10] Huang CC, Chiang HF, Hsieh CC, Lin HC, Wu CH, Lin TM (2023). Percutaneous transluminal angioplasty and stenting of post-irradiated stenosis of subclavian artery: A matched case-control study. J. Neuroradiol..

[11] Gill H, Gill HS, Kotha V (2022). Subclavian atherectomy and angioplasty for coronary subclavian steal syndrome post CABG. Radiol. Case Rep..

[12] European Stroke O, Tendera M, Aboyans V, Bartelink ML, Baumgartner I, Clement D (2011). Esc guidelines on the diagnosis and treatment of peripheral artery diseases: Document covering atherosclerotic disease of extracranial carotid and vertebral, mesenteric, renal, upper and lower extremity arteries: The task force on the diagnosis and treatment of peripheral artery diseases of the European Society of Cardiology (ESC). Eur. Heart J..

[13] Przewlocki T, Kablak-Ziembicka A, Pieniazek P, Musialek P, Kadzielski A, Zalewski J (2006). Determinants of immediate and long-term results of subclavian and innominate artery angioplasty. Catheter. Cardiovasc. Interv..

[14] Chatterjee S, Nerella N, Chakravarty S, Shani J (2013). Angioplasty alone versus angioplasty and stenting for subclavian artery stenosis—A systematic review and meta-analysis. Am. J. Ther..

[15] Gao BL, Xu GQ, Wang ZL, Li TX, Wang YF, Liang XD (2018). Transradial stenting for carotid stenosis in patients with bovine type and type iii aortic arch: Experience in 28 patients. World Neurosurg..

[16] Shen S, Jiang X, Dong H, Peng M, Wang Z, Che W (2019). Effect of aortic arch type on technical indicators in patients undergoing carotid artery stenting. J. Int. Med. Res..

[17] Zhang JL, Tong W, Lv JF, Chi LX (2017). Endovascular treatment and morphology typing of chronic ostial occlusion of the subclavian artery. Exp. Ther. Med..

[18] Schillinger M, Haumer M, Schillinger S, Mlekusch W, Ahmadi R, Minar E (2002). Outcome of conservative versus interventional treatment of subclavian artery stenosis. J. Endovasc. Ther..

[19] Rogers JH, Calhoun RF (2007). Diagnosis and management of subclavian artery stenosis prior to coronary artery bypass grafting in the current era. J. Card. Surg..

[20] Stone PA, Srivastiva M, Campbell JE, Mousa AY (2010). Diagnosis and treatment of subclavian artery occlusive disease. Expert Rev. Cardiovasc. Ther..

[21] Benhammamia M, Mazzaccaro D, Ben Mrad M, Denguir R, Nano G (2020). Endovascular and surgical management of subclavian artery occlusive disease: Early and long-term outcomes. Ann. Vasc. Surg..

[22] Galyfos GC, Kakisis I, Maltezos C, Geroulakos G (2019). Open versus endovascular treatment of subclavian artery atherosclerotic disease. J. Vasc. Surg..

[23] Modarai B, Ali T, Dourado R, Reidy JF, Taylor PR, Burnand KG (2004). Comparison of extra-anatomic bypass grafting with angioplasty for atherosclerotic disease of the supra-aortic trunks. Br. J. Surg..

[24] Schneider V, Dirschinger R, Wustrow I, Muller A, Cassese S, Fusaro M (2020). Endovascular therapy of subclavian artery occlusive disease involving the vertebral artery origin. VASA.

[25] Li Y, Yin Q, Zhu W, Wang Y, Fan X, Liu D (2013). Endovascular stenting for atherosclerotic subclavian artery stenosis in patients with other craniocervical artery stenosis. J. Thromb. Thrombolysis.

[26] Duchman KR, Pugely AJ, Martin CT, Gao Y, Bedard NA, Callaghan JJ (2017). Operative time affects short-term complications in total joint arthroplasty. J. Arthroplast..

[27] Gomez-Hernandez MT, Forcada C, Varela G, Jimenez MF, Spanish Group of Video-Assisted Thoracic Surgery (2022). Operating time: An independent and modifiable risk factor for short-term complications after video-thoracoscopic pulmonary lobectomy. Eur. J. Cardiothorac. Surg..

[28] de Angelis P, Tan KS, Chudgar NP, Dycoco J, Adusumilli PS, Bains MS (2022). Operative time is associated with postoperative complications after pulmonary lobectomy. Ann. Surg..

